# Impact of cocoa flavanol intake on age-dependent vascular stiffness in healthy men: a randomized, controlled, double-masked trial

**DOI:** 10.1007/s11357-015-9794-9

**Published:** 2015-05-27

**Authors:** Christian Heiss, Roberto Sansone, Hakima Karimi, Moritz Krabbe, Dominik Schuler, Ana Rodriguez-Mateos, Thomas Kraemer, Miriam Margherita Cortese-Krott, Gunter G. C. Kuhnle, Jeremy P. E. Spencer, Hagen Schroeter, Marc W. Merx, Malte Kelm, for the FLAVIOLA Consortium, European Union 7th Framework Program

**Affiliations:** Division of Cardiology, Pulmonology, and Vascular Medicine, University Duesseldorf, Medical Faculty, Moorenstr. 5, 40225 Duesseldorf, Germany; Department of Food and Nutritional Sciences, University Reading, Reading, UK; Mars Inc., McLean, VA USA; Cardiovascular Research Institute Duesseldorf, University Duesseldorf, Medical Faculty, Duesseldorf, Germany

**Keywords:** Age, Blood pressure, Arterial stiffness, Endothelial function, Nutrition

## Abstract

Increased vascular stiffness, endothelial dysfunction, and isolated systolic hypertension are hallmarks of vascular aging. Regular cocoa flavanol (CF) intake can improve vascular function in healthy young and elderly at-risk individuals. However, the mechanisms underlying CF bioactivity remain largely unknown. We investigated the effects of CF intake on cardiovascular function in healthy young and elderly individuals without history, signs, or symptoms of cardiovascular disease by applying particular focus on functional endpoints relevant to cardiovascular aging. In a randomized, controlled, double-masked, parallel-group dietary intervention trial, 22 young (<35 years) and 20 elderly (50–80 year) healthy, male non-smokers consumed either a CF-containing drink (450 mg CF) or nutrient-matched, CF-free control drink bi-daily for 14 days. The primary endpoint was endothelial function as measured by flow-mediated vasodilation (FMD). Secondary endpoints included cardiac output, vascular stiffness, conductance of conduit and resistance arteries, and perfusion in the microcirculation. Following 2 weeks of CF intake, FMD improved in young (6.1 ± 0.7 vs. 7.6 ± 0.7 %, *p* < 0.001) and elderly (4.9 ± 0.6 vs. 6.3 ± 0.9 %, *p* < 0.001). Secondary outcomes demonstrated in both groups that CF intake decreased pulse wave velocity and lowered total peripheral resistance, and increased arteriolar and microvascular vasodilator capacity, red cell deformability, and diastolic blood pressure, while cardiac output remained affected. In the elderly, baseline systolic blood pressure was elevated, driven by an arterial-stiffness-related augmentation. CF intake decreased aortic augmentation index (−9 %) and thus systolic blood pressure (−7 mmHg; Clinicaltrials.gov: NCT01639781). CF intake reverses age-related burden of cardiovascular risk in healthy elderly, highlighting the potential of dietary flavanols to maintain cardiovascular health.

## Introduction

**Fig. 1:**
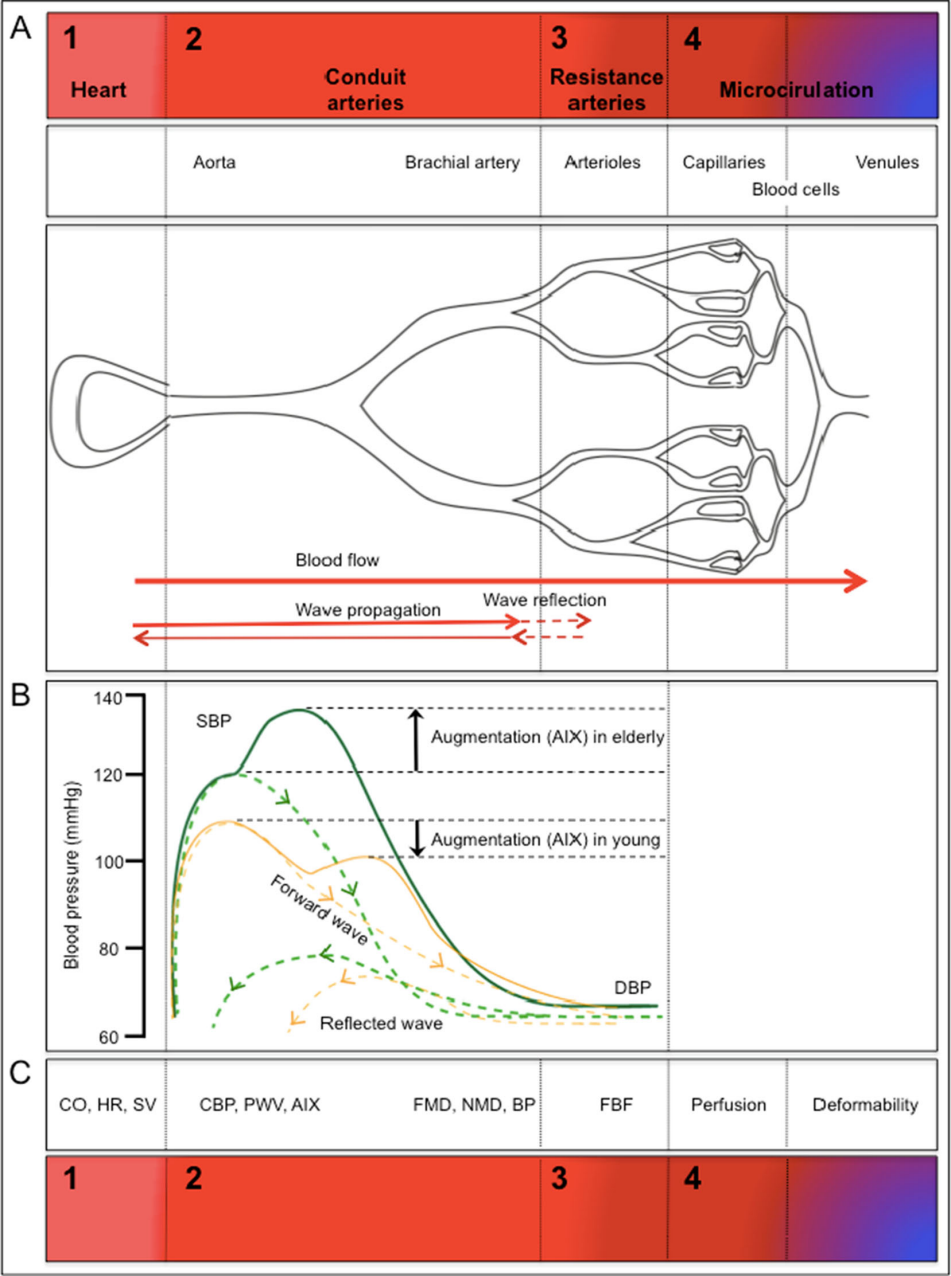
**a** Schematic of cardiovascular system and **b** physiological parameters reflecting basic components of the cardiovascular system. Cardiac function is determined by heart rate (HR), cardiac output (CO), stroke volume (SV), and total peripheral resistance (TPR); the aorta as an elastic artery is characterized by its physicomechanical properties: central blood pressure (BP), pulse wave velocity (PWV), and augmentation index (AIX); conduit artery function is determined by flow-mediated and nitroglycerin-mediated vasodilation (FMD and NMD, respectively) as well as peripheral blood pressure that can be determined at the upper arm and finger; arteriolar conductance is characterized by forearm blood flow (FBF), cutaneous capillary blood flow by laser Doppler perfusion imaging (LDPI), and blood rheology by red blood cell (RBC) deformability

The mechanisms underlying vascular aging are largely unknown but involve all segments of the arterial vascular system. Hallmarks of vascular aging are not entirely identical to those of atherosclerosis, but increasing age is associated with endothelial dysfunction and vascular stiffening, as well as isolated systolic hypertension, and a substantial excess of cardiovascular mortality (Kaess et al. 2012). Due to increased arterial stiffness, older humans exhibit an increased pulse wave velocity (PWV). In young individuals, the reflected pulse wave returns to the heart and increases pressure during diastole and therefore contributes to the “windkessel effect” (i.e., elastic reservoir) of the aorta. In older individuals, the pulse wave propagates faster as compared to the young, and its return to the heart coincides with the systolic forward flow, thus leading to aortic augmentation of systolic blood pressure (SBP; Fig. 1). Furthermore, stiff arteries are characterized by a decreased absorption of the pulse wave, resulting in increased mechanical stress on the arterioles, which in turn is thought to cause damage to the microvasculature, leading to structural microvascular remodeling and rarefication (Mitchell 2008). In addition to an increased peripheral total resistance, age-dependently reduced microcirculatory perfusion, systemic endothelial dysfunction, and intimal hyperplasia are observed in elderly subjects (Celermajer et al. 1994; Rossi et al. 2002). There is great interest in dietary interventions to slow or even reverse the complex pattern of these age-associated vascular alterations, which increase cardiovascular risk (Shai et al. 2010).

Diet is a major determinant of healthy aging, and certain bioactives may be capable of contributing to the maintenance of health, thus extending a healthy life span (Estruch et al. 2013; Williamson et al. 2009). Flavanols, a subgroup of dietary plant-derived bioactives, have gained increasing attention, as clinical dietary interventions have shown that a higher intake of flavanol-containing foods can increase arterial function in individuals at risk for cardiovascular disease (CVD), and in those with established CVD (Heiss et al. 2003; Heiss et al. 2010b). Although the mechanisms of action underlying the biological effects of flavanols are not completely understood, flavanols are one of few bioactives today, for which causality between the dietary intake and an improvement in arterial function has been established (Loke et al. 2008; Schroeter et al. 2006). Whether or not the intake of cocoa flavanol (CF) has the potential to improve cardiovascular function in healthy elderly individuals or to reverse age-related vascular dysfunction has not been specifically evaluated thus far.

The FLAVIOLA research project, funded by the European Union’s 7th framework program, was created to develop the “Targeted delivery of dietary flavanols for optimal human cell function: Effects on cardiovascular health.” In order to understand the hemodynamic mechanisms that underlie the potential CF-intake-mediated reversal of age-associated changes in circulatory function, the present FLAVIOLA AGE study aims at elucidating in great detail the effects of cocoa flavanols in healthy individuals. We therefore studied in young and elderly healthy individuals the effect of a dietary CF intervention on hallmarks of cardiovascular aging in all segments of the cardiovascular system, such as cardiac performance, endothelial dysfunction, increased systolic blood pressure, vascular stiffness, and microcirculatory functions.

## Material and methods

### Study participants and study design

**Fig. 2:**
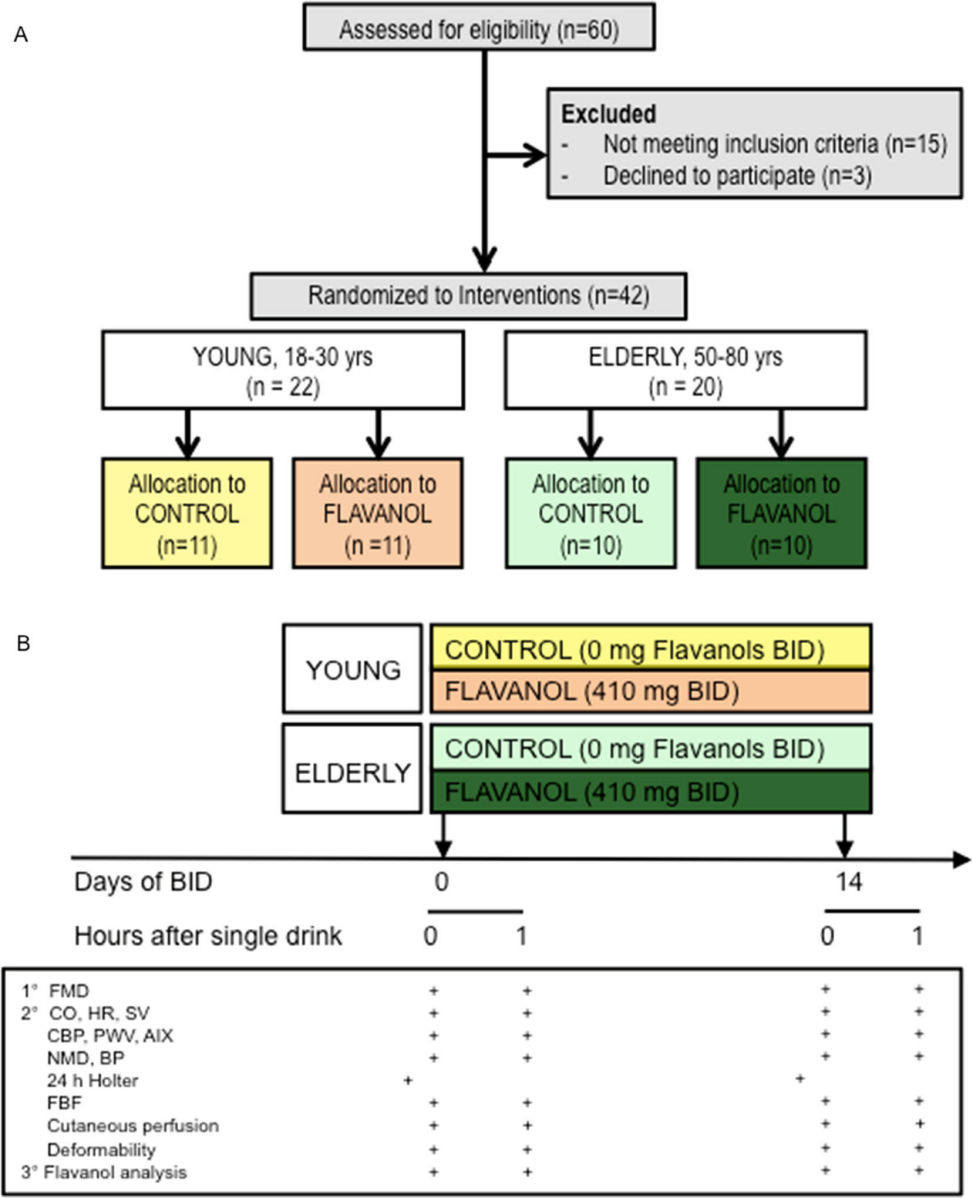
**a** Study flow (CONSORT diagram) and **b** study protocol ll (RBC) deformability

We recruited YOUNG (<35 years) and ELDERLY (50–80 year) healthy male Caucasian adult subjects without history, signs, or symptoms indicative of cardiovascular disease, including previous myocardial infarction, stroke, and peripheral artery disease or current or previous medication (Fig. 2). Participants were randomly assigned to either the CF intake group (FLAVANOL; 450 mg total flavanols two times daily) or a nutrient-matched CF-free group (CONTROL) based on a double-masked, parallel-group study design. All interventions were provided in anonymized sachets. Study participants were instructed to prepare the drinks by emptying the contents of each packet into ∼500 ml of water; drinks were prepared just prior to consumption. Drinks were consume two times per day, one beverage in the morning with breakfast and one with the evening meal. This regimen was maintained for 14 days, with compliance assessed by the collection of empty sachets on the last study day visit.

Measurements were taken fasted before (baseline) and 1 h after the first drink on day 1 and day 14. Endothelial function (primary end point) was measured as flow-mediated vasodilation (FMD). Secondary endpoints included measures of cardiac output, the physicomechanical properties of the large conduit arteries, the dilatory capacity and tone of resistance arteries, and perfusion in the microcirculation (Fig. 1). Tertiary endpoints included total plasma epicatechin metabolites. The study protocol was approved by the ethics committee of the Heinrich-Heine-University; all subjects gave written informed consent (Clinicaltrials.gov: NCT01639781).

### Test materials

Both interventions used a low-calorie fruit-flavored beverage mix (provided by Mars, Inc.), standardized and matched in composition. All beverage mixes were agglomerated powders, utilizing a maltodextrin base into which flavoring and sweeteners were incorporated. The beverage mixes were provided in sachets (7 g, equals one serving) labeled with an alphanumeric identifier to enable a double-masked study design.

A high flavanol cocoa extract (Cocoapro®-processed cocoa extract, Mars Inc.) was the source of flavanols in the CF-containing drink. The CF-containing drink (FLAVANOL) provided 450 mg of total cocoa flavanols per serving (Adamson et al. 1999). The total amount of CF in milligrams represents the sum of all monomeric flavanols and their oligomers (i.e., procyanidins) with a degree of polymerization up to and including 10 (i.e., DP 1–10). The predominant monomeric flavanol in this drink was (−)-epicatechin (see Table 1).

**Table 1:** Baseline characteristics of study population (*t* test, mean [SEM])

		YOUNG	ELDERLY	*p* value
A	n	22	20	
	Age (years)	26 ± 1	60 ± 2	<0.001
	BMI (kg/m^2^)	24.9 ± 0.5	26.5 ± 0.7	0.013
	Height (m)	1.83 ± 0.01	1.81 ± 0.01	0.453
	Weight (kg)	81 ± 2	88 ± 3	0.079
	Creatinine (mg/dl)	1.0 ± 0.03	1 · 0 ± 0.03	0.991
	Total cholesterol (mg/dl)	172 ± 7	207 ± 7	<0.001
	LDL cholesterol (mg/dl)	129 ± 7	157 ± 6	0.005
	HDL cholesterol (mg/dl)	53 ± 4	54 ± 2	0.900
	Triglycerides (mg/dl)	97 ± 44	118 ± 39	0.104
	Fasting plasma glucose (mg/dl)	89 ± 2	95 ± 2	0.027
	Hb_A1c_ (%)	4.8 ± 0.3	4.6 ± 0.4	0.554
	SBP (mmHg)	120 ± 2	131 ± 3	0.006
	DBP (mmHg)	77 ± 2	82 ± 2	0.011
	HR (bpm)	56 ± 2	56 ± 2	0.908
	CRP (mg/dl)	0.1 ± 0.03	0.1 ± 0.03	0.692
	Hb (mg/dl)	15.3 ± 1.0	15.4 ± 1.1	0.721
	Leucocytes (1000/ul)	5.5 ± 0.3	5.8 ± 0.3	0.489
B	Smoking history (*n*)	0	0	
	Medication (*n*)	0	0	
	Hx of CVD (*n*)	0	0	
	Diabetes mellitus (*n*)	0	0	
	Hypercholesterolemia (*n*)	0	6	
	Arterial hypertension (*n*)	0	4	

*SEM* standard error of the mean, *BMI* body mass index, *LDL* low-density lipoprotein, *HDL* high-density lipoprotein, *SBP* systolic blood pressure, *DBP* diastolic blood pressure, *HR* heart rate, *CRP* C-reactive protein, *CVD* cardiovascular disease

The control beverage mix did not contain any cocoa extract and thus provided 0 mg CF (CONTROL). Given the natural presence of theobromine and caffeine in cocoa extract, both theobromine and caffeine were added to the control beverage mix in order to match the composition of alkaloids in the CF-containing test product. Coloring was also added so that the 0 mg CF drink was also indistinguishable in appearance. Compositional details for the 0 mg (CONTROL) and 450 mg CF (FLAVANOL) test drinks are provided in Table 2.

**Table 2:** Composition of interventional vehicles ingested bi-daily (ND = not detectable)

	FLAVANOL	CONTROL
Total cocoa flavanols (mg)	450	ND
Monomers (mg)	73	ND
(−)-Epicatechin (mg)	64	ND
(−)-Catechin (mg)	7	ND
(+)-Catechin (mg)	2	ND
(+)-Epicatechin (mg)	ND	ND
Dimers-decamers (mg)	377	ND
Theobromine (mg)	44	46
Caffeine (mg)	10	6
Fat (g)	0	0
Carbohydrates (g)	6	6
Protein (g)	0.1	0.1
Energy (kcal)	25	25
Sodium (mg)	3	3
Potassium (mg)	95	85

### Hemodynamic monitoring

Cardiac function was determined by heart rate (HR), cardiac output (CO), stroke volume (SV), and total peripheral resistance (TPR); the function of major conduit arteries, elastic aorta and muscular brachial artery, was characterized based on their physicomechanical properties and capacity to dilate (Fig. 1). Physicomechanical properties of the aorta are central blood pressure (BP), pulse wave velocity (PWV), and augmentation index (AIX); dilatory capacity of brachial artery was measured by flow-mediated and nitroglycerin-mediated vasodilation (FMD and NMD, respectively) as well as peripheral blood pressure that was determined at the upper arm and finger; the conductance function of arterioles was evaluated as resting and maximal forearm blood flow (FBF) during reactive hyperemia. Important readouts of microvascular perfusion: Cutaneous perfusion was evaluated by laser Doppler perfusion imaging (LDPI) at rest and maximal perfusion during reactive hyperemia, and blood rheology was determined as red blood cell (RBC) deformability.

### FMD

Brachial artery FMD and nitroglycerin-mediated vasodilation (NMD) were measured by ultrasound (Vivid I, GE) in combination with an automated analysis system (Brachial Analyzer, MIA, Iowa City) as described (Heiss et al. 2010a). Brachial artery (BA) FMD was measured by ultrasound (10-MHz transducer; Vivid I, GE) in combination with an automated analysis system (Brachial Analyzer, MIA, Iowa City, IO) in a 21 °C-temperature-controlled room after 15 min of supine rest. Diameter and Doppler-flow velocity were measured at baseline and immediately after cuff deflation, at 20, 40, 60, and 80 s, and maximal diameter was used to calculate FMD. At the end of each study day, nitroglycerin-mediated vasodilation (NMD) was measured at 4 min after 400 μg sublingual nitroglycerin (Nitrolingual, Pohl).

### Hemodynamic monitoring

The Task Force Monitor (CN-Systems, Graz, Austria) was used for continuous beat-to-beat assessment of cardiovascular variables, including stroke volume, BP, heart rate, and total peripheral resistance by impedance cardiography, which included ECG, phonocardiography, Finapres (Finapres-Medical-Systems, Amsterdam, Netherlands), and BP monitoring system (Dynamap, Tampa, USA).

### BP measurements

Office blood pressure was measured three times after 10 min of rest using an automated clinical digital sphygmomanometer (Dynamap, Tampa, FL, USA) with appropriately sized cuff placed around the upper arm at heart level. Furthermore, we determined 24-h ambulatory BP measurements on the day before day 1 of the study and during the last 24 h of the study on day 13 until the clinical visit on day 14. Values are expressed as day, night, and total average. Furthermore, we determined BP at the fingertip using a tonometric device (Finapres Medical Systems, Amsterdam, Netherlands). Values were taken over 5 min and results represent average of measurements taken. Central BP was derived from peripheral pulse wave analysis obtained with applanation tonometry using a proprietary transfer function (SphygmoCor®, AtCor medical, Australia).

### Pulse wave analysis

Central blood pressure parameters including augmentation index (AIX) were measured by applanation tonometry using the SphygmoCor® system. Via a transfer function, the pressure waveform of the ascending aorta was synthesized. PWV was determined from measurements taken at the carotid and femoral artery as described (Van Bortel et al. 2012).

### Forearm blood flow

Forearm blood flow (FBF) was measured by mercury-in-rubber strain gauge plethysmography (Periquant 833, Gutman, Eurasburg, Germany) according to standard techniques at rest and during reactive hyperemia secondary to 3 min of forearm ischemia.

### Assessment of perfusion in cutaneous microcirculation using laser doppler perfusion imaging

Perfusion of the cutaneous microcirculation of the forearm skin was measured at rest and during reactive hyperemia using a scanning laser Doppler perfusion imager (PeriScan PIM III, Perimed, Sweden) as described (Keymel et al. 2010). The parameter obtained is a global circulatory index expressed in arbitrary units (au) integrating the multidirectional average velocities in skin resistance arteries (lumen <100 m). After 15-min rest, the laser beam was positioned 15 cm above the forearm scanning a field of 200 mm^2^ on the volar site of the forearm. Microvascular reactivity was assessed during postocclusive reactive hyperemia. Following the baseline perfusion (1 min; 20 images), a blood pressure cuff located at the proximal forearm was inflated to suprasystolic pressure. After the cuff release, the microvascular response on reactive hyperemia was recorded. Data acquisition and analysis were performed by LDPIWin Software (Perimed, Sweden). Maximum perfusion, amplitude of perfusion (maximum perfusion − baseline perfusion), ratio (maximum perfusion / baseline perfusion), percentage increase ((maximum perfusion − baseline perfusion) / baseline perfusion × 100), time to peak response, and area under curve were evaluated without subtracting biological zero from the data (Keymel et al. 2010).

### Measurement of RBC deformability

RBC deformability was measured by a laser-assisted optical rotational cell analyzer (LORCA, R&R Mechatronics, Hoorn, Netherlands) according to the manufacturers’ instructions as described (Keymel et al. 2011). After a 15-min rest in a supine position, blood was drawn from the cubital vein, collected in a heparinized tube (10 IE/ml, Liquemin 5000 IE, Roche, Grenzach-Wyhlen, Germany), and RBC deformability was measured by the laser-assisted optical rotational cell analyzer (LORCA, R&R Mechatronics, Hoorn, Netherlands) according to the manufactures’ instructions as described previously. Briefly, 20 μl of whole blood was diluted 200 times in high-viscosity L–polyvinylpyrrollidone. One milliliters of the suspension was transferred into the LORCA device and automatically subjected to varying degrees of shear stress at 0.3 to 10 Pa by stepwise increases of the rotation speed (Keymel et al. 2011).

### Analysis of flavanols and their metabolites in plasma

(−)-Epicatechin and its related metabolites where analyzed in plasma by HPLC-FLD/UV and electrochemical detection using authentic standards provided by Mars Inc., as previously described (Ottaviani et al. 2012). Prior to analysis, plasma samples (0.5 ml) were defrosted on ice and then subjected to β-glucuronidase and sulfatase treatment (2000 units/ml; 40 min; 37 °C). Then, samples were mixed with 2 ml of acidified ice-cold methanol (0.5 % acetic acid in methanol, *v*/*v*) containing 3′-*O*-ethyl-(−)-epicatechin (500 nM) as a recovery standard. Samples were centrifuged at 17,000×*g* for 15 min at 4 °C, and the supernatant was collected. The pellet was extracted again with 2 ml of acidified ice-cold methanol (0.5 % acetic acid in methanol, *v*/*v*) containing 3′-*O*-ethyl-(−)-epicatechin (500 nM), and then with 1 ml of 50 % methanol acidified with 0.5 % acetic acid and containing 3′-*O*-ethyl-(−)-epicatechin (500 nM). Combined supernatants underwent concentration (to approximately 50 μl) using a Speedvac system (Thermo Fisher Scientific Inc., Basingstoke, UK) and were mixed with resorcinol (300 pmol) and catechol (300 pmol) prior to analysis by HPLC. Flavanol monomers and *O*-methylated metabolites were analyzed using a Hewlett-Packard 1200 series HPLC (Hewlett-Packard, Palo alto, CA, USA) equipped with diode array and fluorescent detection. Samples (50 μl) were injected onto a reversed-phase Phenomenex Luna C18(2) column (4.6 × 150 mm) with 3-μm particle size. The mobile phase consisted of (A) HPLC grade water, (B) 200 mM sodium acetate, pH 4.4/Methanol (84/16), and (C) acetonitrile, and the following gradient protocol was run: 0 min, 75 % A, 25 % B; 5 min, 75 % A, 25 % B; 20 min, 65 % A, 25 % B; 28 min, 63 % A, 25 % B; 34 min, 55 % A; 25 % B; 41 min, 45 % A, 25 % B; 45 min, 25 % B, 75 % C; 55 min, 25 % B, 75 % C; 56.1 min 75 % A, 25 % B; and 60 min, 75 % A, 25 % B. The flow rate was 0.8 ml/min. Detection of flavanols and their metabolites was performed using a fluorescent detector with excitation wavelength of 276 nm and emission wavelength of 316 nm. The concentration of each compound was determined using an external calibration curve produced with the use of authentic standards.

### Statistical methods

Results are expressed as mean ± standard error of the mean (SEM). Baseline data represent data of first visit (day 1, 0 h). The primary test for an effect was a three-way repeated mixed model measurements ANOVA (one within-subject factor: time [day 1, 0 h / day 1,1 h / day 14, 0 h / day 14, 1 h]; two between-subject factors: age (YOUNG/ELDERLY) and intervention (CONTROL/FLAVANOL). ANOVA and confidence intervals for pairwise comparisons were computed with SPSS 20 (IBM). Correlations were Pearson’s *r*. Values of *p* less than 0.05 were regarded as statistically significant.

## Results

### Baseline characteristics

A total of 60 male individuals underwent assessment for eligibility [see CONSORT flow diagram (Fig. 2)]; 22 young and 20 elderly subjects were included. None of the participants had been previously diagnosed with, or had clinical signs or symptoms of, cardiovascular disease, such as myocardial infarction, coronary artery disease, hypertension, diabetes, or hyperlipidemia. During the initial diagnostic workup, we observed mild elevations of systolic blood pressure (*n* = 4) and cholesterol (*n* = 6, see Table 1) in a few elderly individuals, which were in a line with the diagnosis of mild isolated systolic hypertension (grade 1) and hypercholesterolemia. Importantly, none of these mild alterations would require medication according to current treatment guidelines (Perk et al. 2012). Compared to the YOUNG, the ELDERLY exhibited statistically significantly greater BMI, total- and LDL-cholesterol, fasting plasma glucose, SBP, and diastolic blood pressure (DBP) at baseline, but all these parameters were within limits considered normal, and none of the subjects had an indication for medical treatment according to current guidelines. The remaining demographic parameters did not significantly differ between groups (Table 1). All subjects completed the study and all data were included in the analysis. The test drinks were well tolerated and no adverse events were reported.

### Baseline hemodynamic characterization of aged vasculature

At baseline on day 1, resting cardiac output was significantly lower in ELDERLY as compared to YOUNG (6.4 ± 0.2 and 5.2 ± 0.3 l/min, *p* < 0.05; Table 3). Given that there was no difference in heart rate (57 ± 3 and 57 ± 2 /min, *p* = n.s.), this was tenably driven by a significantly lower stroke volume in the ELDERY (107 ± 4 and 84 ± 2 ml, *p* < 0.05). Peripheral SBP (121 ± 2 and 128 ± 2 mmHg, *p* < 0.05) and DBP (76 ± 2 and 81 ± 2 ml, *p* < 0.05) as assessed by standard office measurement, as well as total peripheral resistance (TPR), were significantly greater in ELDERLY. Similarly, greater values were observed in 24-h ambulatory Holter monitoring (continuous measurement over 5 min at the fingertip), and central BP. ELDERLY participants exhibited stiffer arteries with significantly greater pulse wave velocity (PWV; 5.9 ± 0.2 and 9.4 ± 0.4 m/s, *p* < 0.05) and aortic augmentation index (AIX; −11 ± 2 and 20 ± 2 %, *p* < 0.05) as compared to YOUNG. Endothelium-dependent and endothelium-independent conduit artery vasodilator function, as measured by FMD (6.3 ± 0.2 and 5.2 ± 0.2 %, *p* < 0.05) and nitroglycerin-mediated (NMD; 15.0 ± 3 and 12.6 ± 0.5 %, *p* < 0.05), respectively, was lower in the ELDERLY. The baseline diameter of the brachial artery was greater in ELDERLY subjects (4.4 ± 0.1 and 4.8 ± 0.2 mm, *p* < 0.05). As detailed in Table 3, resting forearm blood flow (FBF) and laser Doppler perfusion, reflecting primarily skeletal muscle blood flow (arteriolar cross-sectional area) and cutaneous capillary perfusion, respectively, were similar between YOUNG and ELDERLY. However, maximal hyperemic blood flow was lower in ELDERLY as compared to that in YOUNG, suggesting a lessened functional capacity to increase blood flow in response to ischemia. Red blood cell (RBC) deformability was similar in YOUNG and ELDERLY.

**Table 3:**
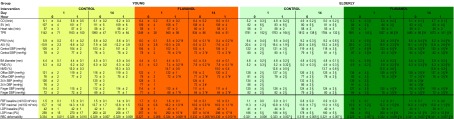
Summary of hemodynamic readouts (Mean [SEM])

*p* < 0.05 versus individual baseline (day 1, 0 h); ^§^ *p* < 0.05 versus YOUNG at the same time point and the same intervention group; ^#^ *p* < 0.05 versus CONTROL in the same age group

### Flavanol intervention improves endothelial function in YOUNG and ELDERLY

The FLAVANOL intervention led to an acute increase in brachial artery FMD at 1 h after CF ingestion in both YOUNG (6.1 ± 0.2 to 7.4 ± 0.2 %, *p* < 0.05) and ELDERLY (4.9 ± 0.2 to 5.9 ± 0.2 %, *p* < 0.05; Table 3). FMD after overnight fast (0 h) on day 14 was higher in both groups (7.8 ± 0.2 and 6.3 ± 0.3 %, *p* < 0.05) as compared to that on study day 1 (0 h). Additional acute ingestion of FLAVANOL did not further increase endothelial function assessed at 1 h on day 14. Despite differences in baseline FMD, it is noteworthy that each individual exhibited positive responses following FLAVANOL intake. The mean changes in FMD following FLAVANOL were 1.3 ± 0.6 % in the young and 1.0 ± 0.6 % in the elderly at 1 h and 1.6 ± 0.8 % in the young and 1.4 ± 0.7 % in the elderly at 14 days. To evaluate determinants of inter-individual response, we correlated the change in FMD at 1 h and 14 days with baseline epidemiological parameters (age, height, weight, BMI, total cholesterol, LDL, HDL, office SBP, office DBP). This analysis showed that none of these parameters correlated with changes in FMD. The baseline diameter of the brachial artery and NMD remained unaffected by either FLAVANOL or CONTROL.

### Flavanol intervention decreases SBP in ELDERLY and this is linked to decreased arterial stiffness and augmentation index

As depicted in Table 3, our results show that FLAVANOL, but not CONTROL, led to a significant *acute* decrease in PWV in ELDERLY (9.3 ± 0.5 to 8.6 ± 0.5 m/s, *p* < 0.05) and YOUNG (6.0 ± 0.1 to 5.5 ± 0.2 m/s, *p* < 0.05) and that PWV remained decreased at 0 h on day 14 (5.6 ± 0.1 and 8.5 ± 0.4 m/s, *p* < 0.05) and did not further decrease at 1 h after FLAVANOL drink at day 14. Importantly, FLAVANOL only decreased office peripheral SBP (acute, −5 mmHg; chronic, −6 mmHg; *p* < 0.05) and AIX (acute, −6 %; chronic, −7 %; *p* < 0.05) in ELDERLY, and this was detectable at all time points investigated as compared to day 1, 0 h. Notably, this decrease in SBP was confirmed by 24-h ambulatory monitoring and central blood pressure measurements. We found a significant correlation between the changes in SBP and AIX after FLAVANOL (*r* = 0.77, *p* = 0.009). It is known that part of the PWV can be modulated by endothelial function. It is noteworthy that the changes in PWV showed a close correlation with improvements in FMD at day 14 (*r* = −0.56, *p* = 0.01), thereby corroborating a mechanistic link between increases in endothelial function and a decrease in SBP in ELDERLY subjects.

### Flavanol intake decreases systemic vascular resistance

As detailed in Table 3, following acute consumption of FLAVANOL, DBP and TPR significantly decreased in both groups, and both parameters remained decreased at day 14, whereas cardiac hemodynamics—cardiac output, heart rate, and stroke volume—were not affected. At all time points after ingestion of FLAVANOL, qualitatively identical changes were observed in DBP on the level of central, brachial artery, and finger BP. These in-office measurements were confirmed by 24-h ambulatory BP monitoring. No significant effects were observed after CONTROL drink. An increased maximal FBF in both groups suggests a functional improvement in arteriolar blood flow capacity. A significant univariate correlation existed between DBP decrease and increase in maximal FBF(*r* = −0.6, *p* = 0.01). This suggests that the FLAVANOL intervention acted primarily on the vascular and not the cardiac component of systemic hemodynamics.

### FLAVANOL intervention decreases RBC deformability

RBC deformability increased after FLAVANOL intervention in both YOUNG and ELDERLY. This was paralleled by significantly increased maximal perfusion of the cutaneous capillaries as measured by laser Doppler during reactive hyperemia. Significant univariate correlation existed between the increase in maximal perfusion and RBC deformability (*r* = 0.6, *p =* 0.01).

### Levels of structurally related flavanol metabolites

At the baseline visit, only low levels of total plasma flavanols were detected (YOUNG, 21 ± 2 nM; ELDERLY, 32 ± 2 nM). At 1 h (day 1) after acute consumption of FLAVANOL, but not CONTROL, both, YOUNG and ELDERLY, showed significantly increased levels of plasma flavanols (668 ± 19 and 704 ± 29 nM, respectively; *p* < 0.0001 with respect to baseline; *p >* 0.05 between young and elderly). After 14 days of two-times-daily CF consumption and following overnight fasting, baseline levels remained significantly higher as compared to those on day 1. Additional acute ingestion of FLAVANOL led to flavanol plasma levels of 745 ± 21 and 857 ± 50 nM (*p <* 0.0001 vs. baseline day 14) in YOUNG and ELDERLY, respectively, but these levels were not significantly different from values observed after acute ingestion on day 1.

## Discussion

The *FLAVIOLA AGE* study is the first study specifically intended to investigate CF-associated improvements of hallmarks of cardiovascular aging, on the level of cardiac performance, endothelial dysfunction, increased systolic blood pressure, vascular stiffness, and microcirculatory functions. The outcomes demonstrate how a CF-based dietary intervention affects the cardiovascular system and reverses age-related increases of blood pressure together with vascular stiffness in healthy elderly humans. CF-intake-associated improvements in the compliance of large arteries were accompanied by a lowered pulse wave velocity and decreased aortic augmentation of systolic blood pressure. Endothelial function in large conduit arteries was significantly improved in healthy young and elderly individuals. These beneficial effects were associated with an improved dilatory capacity of resistance arteries, decreased diastolic blood pressure, and increases in microcirculatory perfusion and red blood cell deformability. These CF-intake-mediated modulations of circulatory function were independent of changes in cardiac performance, since cardiac output was not affected by CF (Table 3).

### CF intake improves endothelial function in young and elderly

Consistent with previous studies in other populations, ELDERLY exhibited significantly decreased endothelium-dependent vasodilation at baseline as compared to YOUNG (Heiss et al. 2005). So far, no study has specifically investigated the vascular effects of CF in healthy elderly and compared it to young individuals. CF-intake-related improvements in FMD occurred acutely following intake but, more importantly, were also manifest when comparing baseline FMD (fasted; 0 h) on days 1 and 14. Moreover, and despite differences in baseline FMD, our results show that the absolute effect size of FMD improvements was similar in young and elderly healthy men. It is also noteworthy that the bioavailability of flavanols in the healthy young men was comparable to that in elderly individuals, as evidenced by flavanol plasma concentrations.

### CF intake attenuates age-related increase in blood pressure and arterial stiffness

Our results show that the CF-intake-dependent decrease in BP in ELDERLY was selective to SBP (Table 3). An endothelial function increase along with a decrease in PWV was also present in the YOUNG, but as would be expected from the initial absence of BP augmentation in this group, these changes did not result in a modulation of SBP. A similar relationship between baseline BP and the effect size of BP-lowering therapeutic agents, which is in a similar range as shown here for flavanols, has been reported for various drugs, including ACE inhibitors (O’Rourke and Hashimoto 2007). Tenable explanations of how flavanols could affect age-related changes in SBP and PWV may be found in the observation of improved compliance due to improved endothelial function as well as decreases in DBP, and thus changes in the distal reflection point of the pulse wave.

### CF intake decreases diastolic blood pressure and increases conductance of small arteries

We observed significant decreases in DBP and also in TPR in both young and elderly participants. CFs (30–1080 mg total flavanols per day) have been shown to elicit a small, but significant DBP-reducing effect of −2.2 mmHg in a meta analysis of a heterogeneous study population including patients with and without CVD (Ried et al. 2012). Resistance to blood flow is principally mediated by arteriolar diameter, which is modified by arteriolar vasoconstriction and dilation, respectively. We observed a significant increase in the maximal forearm blood flow and cutaneous perfusion during reactive hyperemia. This suggests that CF intake may increase not only the dilatory function of conduit arteries but also that of the arterioles. This effect, together with the increased RBC deformability, contributes to the enhanced perfusion of the microcirculation observed following CF intake. Mechanistically, CF-related vascular effects have been linked to an increase in nitric oxide synthase (NOS) activity (Del Rio et al. 2013; Schroeter et al. 2006). RBCs have been demonstrated to express a functional eNOS that may modulate RBC deformability independent of vascular tone (Cortese-Krott et al. 2012; Horn et al. 2011). Consequently, our current data support the supposition that CF intake improves tissue perfusion, at least in part, by increasing dilatory capacity of arterioles and increasing RBC deformability.

### The FLAVIOLA AGE study and healthy vascular aging

Aging is considered to be an independent cardiovascular risk factor in most methods of CV risk scoring. This raises the important question as to whether or not the regular intake of vasculoprotective bioactives not only does reverse age-associated changes of circulatory function but also has the potential to maintain vascular function with increasing age. It is tempting to speculate that such an approach may ultimately support healthy vascular aging, extend the healthy life span, and lead to a lower rate of cardiovascular events. While such a proposition could only be finally verified through long-term longitudinal studies in healthy elderly individuals, it is possible to meaningfully advance our understanding of this question by way of smaller-scale investigations like the FLAVIOLA AGE study, which can provide the necessary basis for larger-scale studies in future. The present study extended our knowledge regarding the potential role of CF intake in the maintenance of optimal cardiovascular function during normal aging. We demonstrated here that CF intake is effective not only in healthy young but also in elderly individuals, as evidenced by the CF-related reversal of age-specific alterations in circulatory function, vascular stiffness, and hemodynamics.

## Limitations

Intending to specifically focus on mechanistic aspects in greater detail than can be done practically in a larger-scale and longer-term study, the duration of this study was chosen to be 2 weeks. This is based on the outcomes of our previous work showing that approximately 2 weeks of CF intake at 450 mg two times daily is sufficient to establish maximal and sustained improvements (plateau) in FMD in this study population. Consequently, it can be argued tenably that despite of limitations with regard to study duration, our data are relevant in furthering understanding of pharmacodynamics aspects of CF-mediated changes in endothelial function, cardiac output, conductance of conduit and resistance arteries, and perfusion in the microcirculation. An additional limitation regarding the design of this study is the exclusion of female participants. The rationale for doing so was based on complexities related to the influence on FMD and other cardiovascular readouts of hormonal changes that occur in women during the menstrual cycle and during peri-menopause and menopause. To assess detailed mechanistic aspects of CF-intake-related changes in cardiovascular function over the background of menstrual-cycle-dependent modulations, it will require a specific and comprehensive study design, which was beyond the scope of this investigation. Finally, the elderly subjects in our study presented with significantly lower cardiac output as compared to the young subjects that was not significantly changed by the flavanol intervention. It has been appreciated for a long time that besides stiffening of the arteries, stiffening of the heart occurs with advancing age (Tresch and McGough 1995), leading to diastolic dysfunction (Upadhya et al. 2015). Whereas a lack of flavanol-related effects on cardiac output may be interpreted as a lack of major flavanol effect on cardiac function, more detailed studies with echocardiography and potentially spiro-ergometry are needed to investigate the impact of flavanols on diastolic function and cardiac performance capacity.

## Conclusions

A CF-based dietary intervention in elderly individuals decreases systolic blood pressure and improves vascular stiffness. These beneficial effects are associated with improved endothelial function in large arteries and enhanced vasodilator function in resistance arteries, as well as with improved microcirculatory perfusion that can already be observed in young healthy participants. Our findings provide clear evidence and novel insights that scientifically underpin the potential of dietary CF to reverse, or at least to attenuate, features of circulatory dysfunction associated with vascular aging in healthy men, a major contributor to atherogenic risk independent of numeric age.
